# Higher Trophic Levels Overwhelm Climate Change Impacts on Terrestrial Ecosystem Functioning

**DOI:** 10.1371/journal.pone.0136344

**Published:** 2015-08-20

**Authors:** Shannon L. Pelini, Audrey M. Maran, Angus R. Chen, Justine Kaseman, Thomas W. Crowther

**Affiliations:** 1 Department of Biological Sciences, Bowling Green State University, Bowling Green, OH, 43403, United States of America; 2 Harvard Forest, Harvard University, Petersham, MA, 01366, United States of America; 3 School of Forestry and Environmental Studies, Yale University, New Haven, CT, 06511, United States of America; Swedish University of Agricultural Sciences, SWEDEN

## Abstract

Forest floor food webs play pivotal roles in carbon cycling, but they are rarely considered in models of carbon fluxes, including soil carbon dioxide emissions (respiration), under climatic warming. The indirect effects of invertebrates on heterotrophic (microbial and invertebrate) respiration through interactions with microbial communities are significant and will be altered by warming. However, the interactive effects of invertebrates and warming on heterotrophic respiration in the field are poorly understood. In this study we combined field and common garden laboratory approaches to examine relationships between warming, forest floor food web structure, and heterotrophic respiration. We found that soil animals can overwhelm the effects of warming (to 5 degrees Celsius above ambient) on heterotrophic respiration. In particular, the presence of higher trophic levels and burrowing detritivores strongly determined heterotrophic respiration rates in temperate forest soils. These effects were, however, context-dependent, with greater effects in a lower-latitude site. Without isolating and including the significant impact of invertebrates, climate models will be incomplete, hindering well-informed policy decisions.

## Introduction

There is a critical need to improve estimates of future soil CO_2_ emissions (soil respiration, R_S_), which are 10 × greater than those generated by the burning of fossil fuels [1], and the potential feedbacks of the emissions to climate change. The impacts of increasing temperatures on microbes, which, along with plant roots are the primary contributors to R_S_, are included in determination of soil-climate change feedbacks [2–5]. While the direct contribution of other soil and litter-dwelling organisms (e.g., invertebrate detritivores, vertebrate predators) to the heterotrophic component of R_S_ (i.e., R_H_) is presumed to be small relative to that of microbes, the indirect effects of climate change on R_H_ through animal-microbe interactions have not been sufficiently explored. Indeed, the absence of soil animals, particularly invertebrates, has been highlighted as a major limitation to current climate and carbon cycling models and management practices [6–10].

Soil food webs are dominated by invertebrates, which govern decomposition rates and soil structure via their interactions with microbial communities [11–17]. Direct trophic effects of invertebrates can regulate microbial activity in some regions [18,19], but the indirect effects generally enhance microbial growth and respiration. That is, by shredding litter [15,17,20,21] and moving soil [11,12,22,23], invertebrate engineers have the potential to stimulate microbial growth and nutrient mineralization [24].

Temperate forests harbor a variety of organisms that also have the capacity to drive cascading effects on microbial activity. However, there is mixed support for top-down trophic cascades on R_H_ via invertebrate predation [25,26]. Best and Welsh [27] demonstrated that salamanders affect forest leaf litter retention by regulating invertebrate populations, but the impacts on microbes and R_H_ remain unknown. The potential for cascading effects on R_H_ needs to be explored further because multiple studies have shown that warming strengthens the effects of predators on lower trophic levels and nutrient cycling in other food webs [21,28,29].

Invertebrates are highly responsive to climatic change [reviewed in 30]. Because invertebrates are ectotherms, the rates and magnitudes of predation and engineering should increase with temperature, at least until thermal thresholds are exceeded or competitive interactions shift [31]. The effects of warming on invertebrate activity may be magnified if soil invertebrates increase movement throughout the soil [32]. Based on this pattern, our understanding of R_H_ responses to warming remains incomplete until we consider the indirect effects of warming mediated through invertebrates.

In this study we examined relationships between warming, forest floor food web structure, and R_H_. We combined field and common garden laboratory experiments to separate indirect from direct and individual from community structure responses to warming on R_H_. More specifically, using open-top warming chambers at Harvard Forest and Duke Forest, we evaluated the effects of (1) warming on food web structure-R_H_ interactions, (2) macroinvertebrate communities shaped by different warming scenarios on R_H_, and (3) soils and their associated microbial and smaller invertebrate communities (micro/mesofauna) shaped by different warming scenarios on R_H_. Because we placed the same number of animals in all warming chambers, the field experiment (1) allowed us to assess how behavioral or physiological responses to warming affected R_H_. In contrast, we did not manipulate fauna in the common garden experiments (2 and 3), allowing us to determine if communities—macroinvertebrates (2) or micro/mesofauna (3)—shaped by warming differ in R_H_ even when the direct effects of warming on R_H_ were removed.

## Methods

### Study System

Access to the Harvard Forest field sites was permitted by Harvard University, and Duke University granted access to the Duke Forest field site. Use of salamanders was reviewed and approved by Harvard University/Faculty of Arts & Sciences Standing Committee on the Use of Animals in Research & Teaching (IACUC), approved Animal Experimentation Protocol No. 12–16.

We used open-top warming chambers and forest floor communities within or near them at Duke Forest and Harvard Forest [33]. Each of these two sites is equipped with 12 chambers (5 m diameter): 3 are ambient controls while the remaining 9 are heated, through forced air delivery, to different degrees ranging from 1.5–5.5°C above ambient air temperature. These chambers had been running for three years when we began this study.

### Field mesocosms (Experiment 1)

We manipulated dominant components of the forest floor food webs within mesocosms (19 L buckets, 30 cm diameter, 44 cm height) placed in Harvard Forest warming chambers. We filled each mesocosm to 10 cm height with topsoil (O and A horizons, Canton loam) that we collected ~100–200 yards away from the chambers, from which we removed roots and macrofauna (e.g., beetles, ants, spiders, millipedes) via sieving (1mm mesh), and homogenized across all mesocosms.

Biota treatments included 1: no fauna present; 2: only micro/mesofauna present; 3: only macroinvertebrates present; 4: micro/mesofauna and macroinvertebrates present; and 5: micro/mesofauna, macroinvertebrates, and a vertebrate predator present. We removed micro- (e.g., fungi, bacteria) and meso- (e.g., mites, springtails) fauna (Treatments 1 & 3) by autoclaving soil described above at 120°C for 30 minutes. We used red back salamanders (*Plethodon cinereu*) as the top predator in this experiment (Treatment 4) because salamanders are the most abundant vertebrates in many forest ecosystems [34] and have been shown to impact nutrient cycles in deciduous forests [27]. For treatments that included macroinvertebrates (Treatments 3 & 4), we used ~1.5 g each of live earthworms (Megadrilacea), a common invertebrate engineer and food source for salamanders, and mealworms (Tenebrionidae), one of the most abundant macroinvertebrates in the warming chambers at the time of experimental setup.

Into each mesocosm, we also installed a 10 cm diameter, 4.5 cm high, PVC collar, inserted approximately 1 cm into the soil. Within these collars we collected weekly soil efflux (R_H_) measurements using a LiCor 6400 (Lincoln, Nebraska). We counted and weighed all surviving macroinvertebrates at the end of the experiment. Mesocosms were present in the chambers for 6 weeks during June-August 2013.

### Common garden mesocosms (Experiments 2 & 3)

#### Experiment 2: Macroinvertebrates

We collected litter and organic soil cores (20 cm diameter, 5–8 cm depth) from open-top warming chambers at Harvard Forest and Duke Forest [see 33] in June. We extracted macroinvertebrates, which we identified to class or order, from these cores using 48 hr Berlese funnel extraction and hand removal. We placed these live extracted invertebrates into PVC mesocosms (8 cm diameter, 10 cm depth; herein “macrofauna mesocosms”) in Harvard Forest greenhouse facilities. Mesocosms contained sieved, homogenized organic soil collected adjacent to the warming chambers at Harvard Forest.

#### Experiment 3: Micro/mesofauna

We collected additional soil cores from Harvard Forest chambers to determine if soils, along with micro- and mesofauna, originating from different warming scenarios varied in R_H_ when placed into common conditions. We extracted four organic soil cores (20 cm diameter, 5–8 cm depth) from each chamber and placed them into PVC mesocosms (8 cm diameter, 10 cm depth herein “mico/mesofauna mesocosms”) in Harvard Forest greenhouse facilities.

We have data from Harvard Forest macrofauna mesocosms for two weeks, Duke Forest macrofauna mesocosms for four weeks (Experiment 2), and Harvard Forest micro/mesofauna for six weeks (Experiment 3). From all mesocosms we collected weekly soil efflux (R_H_) measurements and macroinvertebrate and microbial C biomass at the end of the experiment. We used a LiCor 6400 (Lincoln, Nebraska) for soil respiration measurements. We used chloroform fumigation to estimate microbial C [modified from 35].

### Data analyses

#### Field Experiment

We used linear mixed effects modeling to determine if R_H_ varied significantly with warming (difference between average air temperature in heated versus control chambers during the experiment), biota treatments (none; micro/mesofauna only; macroinvertebrates only; micro/mesofauna + macroinvertebrates; micro/mesofauna + macroinvertebrates + salamander), and their interactions with time or each other (fixed effects) in mesocosms (nested within chamber; random effect). We accounted for repeated measures by including a time correlation structure in the model.

For mesocosms with macroinvertebrates, we used linear mixed modeling to determine if biota treatment, warming, and/or their interaction (fixed effects) were significantly associated with macroinvertebrate mortality. For these mesocosms, we also regressed the final R_H_ measurements against macroinvertebrate biomass.

#### Common garden experiments

We pooled across the four soil cores collected in each warming chamber to determine if inter-chamber variation in average air temperature and soil moisture (April 2010 to June 2013; herein ‘microclimate’) was associated with macroinvertebrate community structure. We used linear regression to determine if taxonomic richness or abundance, and PERMANOVA to determine if community composition, varied with chamber microclimate. We did not pool across soil cores for analyses of mesocosm R_H_ because fauna extracted from each soil core were held in separate mesocosms from which R_H_ was measured. We used linear mixed models to determine if R_H_ varied with microclimate in warming chambers from which fauna were collected, microbial biomass (for micro/mesofaua mesocosms and Duke Forest macrofauna mesocosms), and abundance of common macroinvertebrates. We modeled mesocosm as a random effect and incorporated time into the correlation structure of our models to account for repeated measures.

We performed all analyses in R (version 3.0.2). We obtained P-values via likelihood ratio tests of full models compared to those lacking the effects of interest. All raw data are available on the Harvard Forest data archive [36].

## Results

### Field experiment

**Fig 1:**
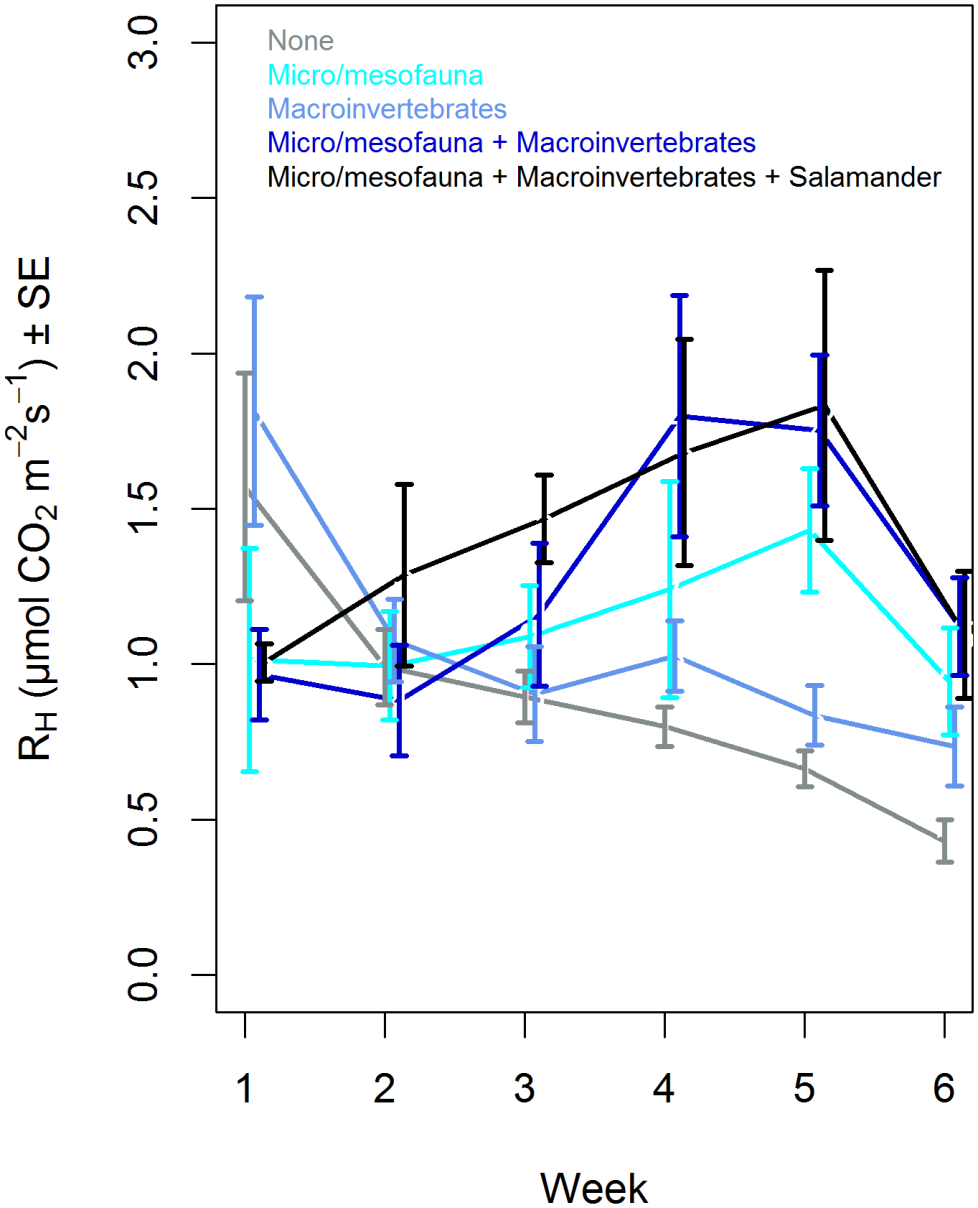
Average field mesocosm R_H_ (μmol CO_2_ m-2 s-1 ± SE), through time, for biota treatments, averaged across warming treatments.

The effect of the biota treatments on R_H_ varied with time (*L*(15) = 21; P = 0.001). In the first week of the experiment, R_H_ was highest in micro/mesofauna removal mesocosms, but in later weeks R_H_ in these mesocosms was lower than that in the other mesocosms (Fig 1). Warming and its interactions with biotic treatment or time were not significant predictors of R_H_.

**Fig 2:**
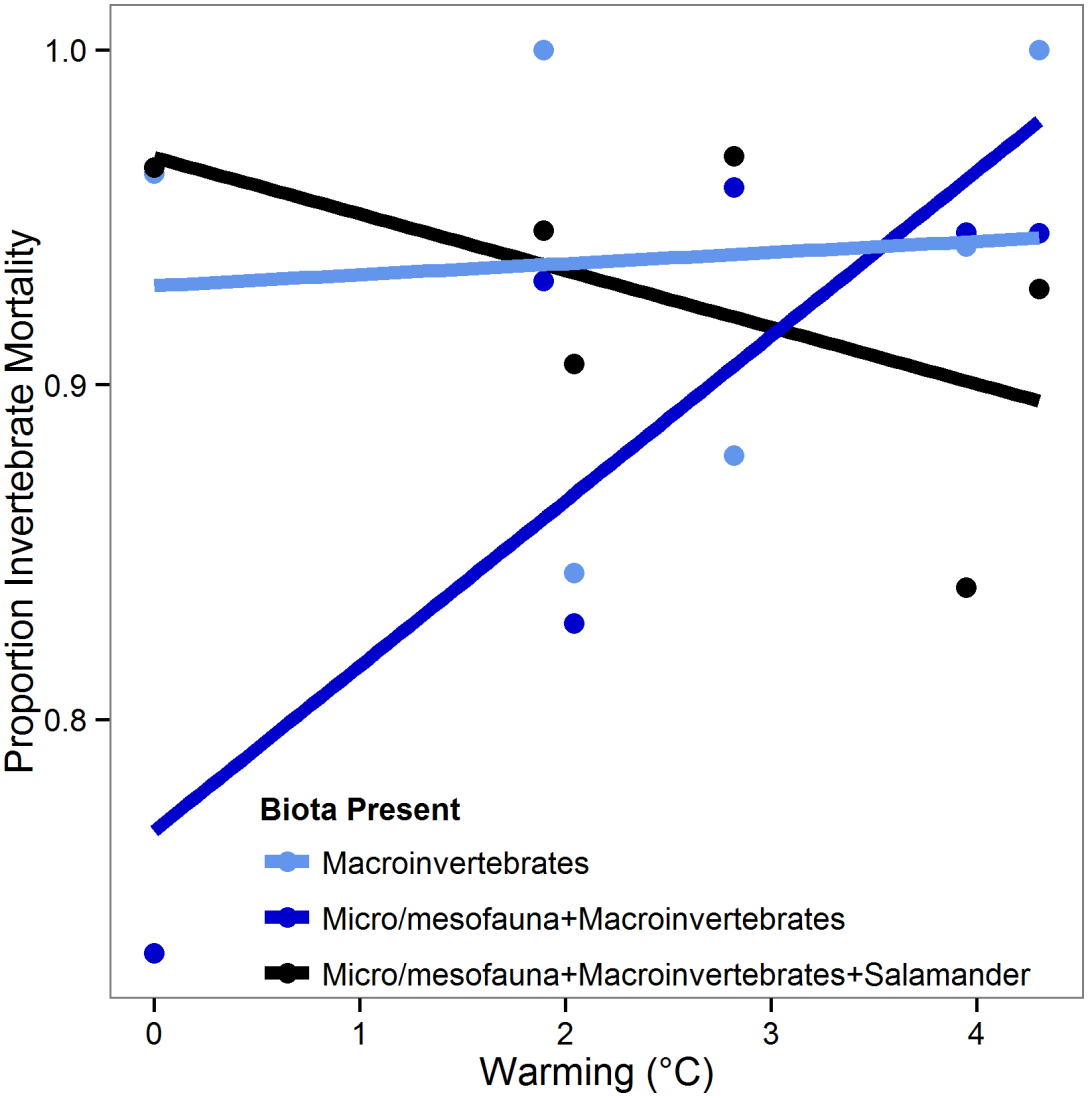
Proportion invertebrate mortality, in biota treatments with macroinvertebrates, across warming treatments.

The impact of warming on macroinvertebrate mortality varied with biota treatment (*L*(7) = 9.5; P = 0.009). More specifically, macroinvertebrate mortality did not vary with warming when micro/mesofauna and salamander predators were absent. However, the addition of micro/mesofauna yielded a strong positive association of warming and invertebrate mortality, but only when salamanders were not present (Fig 2).

### Common garden

**Fig 3:**
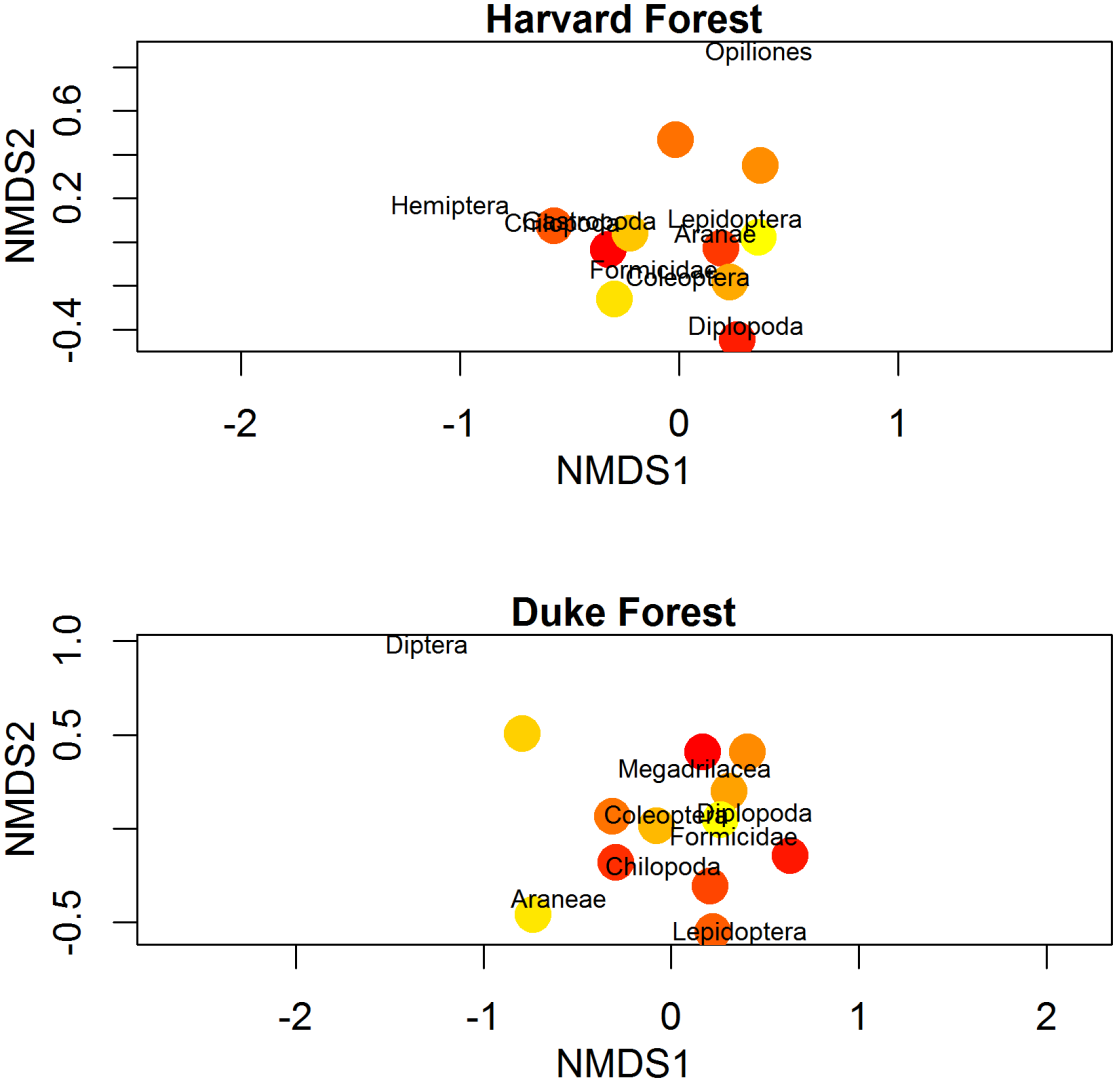
NMDS plot for macroinvertebrate communities extracted from soil cores collected at Harvard Forest (top panel) and Duke Forest (bottom panel) warming chambers. The colors represent the average air temperature in chambers April 2010-June 2013; the yellow to red color gradient represents cooler to warmer chambers.

We extracted Araneae (spiders), Diplopoda (millipedes), Chilopoda (centipedes), Diptera (fly larvae), Coleoptera (beetles), Formicidae (ants), Lepidoptera (caterpillars), and Megadrilacea (earthworms) from Duke Forest and Harvard Forest warming chamber soil cores. Additionally, we collected Gastropoda (slugs) and Opiliones (Harvestmen) from Harvard Forest cores. Macroinvertebrate taxonomic richness and abundance did not vary significantly with microclimate in Duke Forest (F_2,9_ = 0.16, p = 0.85) or Harvard Forest (F_2,9_ = 4.48, p = 0.067) warming chambers. Community composition was not strongly associated with microclimate at either site (PERMANOVA: HF_temperature_ F_1,7_ = 0.55, P = 0.73; HF_moisture_ F_1,7_ = 0.89, P = 0.50; DF_temperature_ F_1,9_ = 1.1, P = 0.39; DF_moisture_ F_1,9_ = 1.6, P = 0.17; Fig 3).

R_H_ of micro/mesofauna mesocosms from Harvard Forest warming chambers did not vary significantly with microclimate of origin or microbial biomass (temperature: *L*(7) = 0.021 p = 0.89; microbial biomass: *L*(7) = 0.26, p = 0.61; moisture: *L*(7) = 0.61, p = 0.44). Similarly, R_H_ of macrofauna mesocosms from Harvard Forest warming chambers did not vary significantly with any microclimate or fauna parameters (Table 1). In contrast, R_H_ in Duke Forest macrofauna mesocosms increased significantly with abundance of millipedes and microbial biomass (Table 2).

**Table 1:** Model results for Harvard Forest macrofauna common garden mesocosms. Also listed are the range of values observed across treatments.

Parameter	Min-Max	df	*L*	P
Air temperature in original warming chamber	9.0–14.0°C	11	0.006	0.94
Soil moisture in original warming chamber	0.11–0.19 VWC	11	0.074	0.78
Coleoptera abundance	7–18	11	0.22	0.64
Aranae abundance	1–7	11	0.87	0.35
Formicidae abundance	1–20	11	0.001	0.97
Diplopoda abundance	0–17	11	0.54	0.46
Taxonomic richness	4–7	11	0.36	0.55

**Table 2:** Model results for Duke Forest macrofauna common garden mesocosms.

Parameter	Min-Max	df	*L*	P
Air temperature in original warming chamber	15.0–21.5°C	12	2.9	0.089
Soil moisture in original warming chamber	0.11–0.28 VWC	12	2.1	0.14
Microbial biomass	204–283 μg C /g	12	9.5	0.002
Megadrilacea abundance	0–10	12	3.2	0.072
Formicidae abundance	0–8	12	0.91	0.34
Coleoptera abundance	4–17	12	0.15	0.70
Aranae abundance	0–3	12	0.14	0.71
Diplopoda abundance	0–7	12	4.5	0.035
Taxonomic richness	3–6	12	0.17	0.68

## Discussion

Our study highlights the prominent role of soil communities in the functioning of temperate forest ecosystems. In particular, it shows how initial differences in soil communities can overwhelm the direct effects of climate change on carbon exchanges between terrestrial and atmospheric pools. These effects were, however, context-dependent, with greater effects in a lower-latitude site. The context-dependent effects of soil fauna are consistent with the results of a previous global litter decomposition study [21], and highlight that the strength of the link between soil communities and nutrient cycling is likely to increase at lower latitudes, where activity levels are not limited by temperature or moisture availability.

The primary drivers of the changes in R_H_ were the epigeic (litter dwelling) and endogeic (burrowing) macrodetritivores (i.e., millipedes, earthworms). Via the comminution of litter and the mixing of soil, these taxa are known to influence the activity of soil microbes and stimulate the mineralization (i.e., efflux, or respiration) of carbon in soil [23,24,37]. We found that the addition of larger detritivores, regardless of warming treatment, altered R_H_. However, despite similar invertebrate densities at both sites, the effects of millipede and earthworm abundance on R_H_ were apparent only in the low latitude site (Duke Forest) when placed in a common garden. Perhaps the burrowing taxa at these two sites represent different species pools that vary in behaviors related to feeding, burrowing, and redistributing soil organic matter in ways that yield higher R_H_ [37,38].

Although ecosystem models [e.g., 2] generally suggest that soil communities are controlled predominantly by bottom-up forces, a growing body of work highlights the potential of trophic interactions to limit microbial activity [14,19,20,39,40]. When microbes are not limited by temperature, top-down control emerges as an important regulatory force [10]. Multiple studies have shown that warming strengthens the effects of predators on lower trophic levels and nutrient cycling in other systems [21,28,29]. In some cases these invertebrate impacts are equal to or greater than the direct effects of warming on nutrient cycling [41]. In our study, warming did not alter the composition of macroinvertebrate communities, but the response of burrower mortality to warming varied with food web structure. Macroinvertebrate mortality was more variable, and responsive to warming, when multiple trophic groups were present.

Our work highlights that, although the direct effects of climate change have the potential to influence the functioning of terrestrial ecosystems, the indirect effects—mediated through changes in the biological community—are likely to be more important in governing soil carbon emissions. This finding supports a growing body of work in aboveground ecology, showing that the indirect effects of change in the biological community can overwhelm the initial direct effects of climate change [40,42,43]. However, our work suggests that warming alone is unlikely to drive these changes. It is important to explore the interacting effects of other global change factors (e.g. N addition and land use change) to understand fully the impacts of anthropogenic activity for the functioning of temperate forests.
